# Use of the PCR-DGGE Method for the Analysis of the Bacterial Community Structure in Soil Treated With the Cephalosporin Antibiotic Cefuroxime and/or Inoculated With a Multidrug-Resistant *Pseudomonas putida* Strain MC1

**DOI:** 10.3389/fmicb.2018.01387

**Published:** 2018-06-26

**Authors:** Kamila Orlewska, Zofia Piotrowska-Seget, Mariusz Cycoń

**Affiliations:** ^1^Department of Microbiology and Virology, School of Pharmacy with the Division of Laboratory Medicine, Medical University of Silesia, Sosnowiec, Poland; ^2^Department of Microbiology, University of Silesia, Katowice, Poland

**Keywords:** cefuroxime, multidrug resistance, *Pseudomonas putida*, DGGE, microbial diversity, soil, multivariate analysis

## Abstract

The widespread use of cefuroxime (XM) has resulted in the increase in its concentration in hospital and domestic wastewaters. Due to the limited removal of antibiotics and antibiotic-resistant genes in conventional systems, the drugs enter the surface water and soils. Moreover, the introduction of XM and/or XM-resistant bacteria into soil may cause a significant modification of the biodiversity of soil bacterial communities. Therefore, the goal of this research was to assess the genetic diversity of a bacterial community in the cefuroxime (XM1 – 1 mg/kg and XM10 – 10 mg/kg) and/or antibiotic-resistant *Pseudomonas putida* strain MC1 (Ps – 1.6 × 10^7^ cells/g)-treated soils as determined by the DGGE (denaturing gradient gel electrophoresis) method. The obtained data were also evaluated using a multivariate analysis and the resistance (RS)/resilience (RL) concept. Strain MC1 was isolated from raw sewage in the presence of XM and was resistant not only to this antibiotic but also to vancomycin, clindamycin and erythromycin. The DGGE patterns revealed that the XM10 and XM10+Ps treatments modified the composition of the bacterial community by the alteration of the DGGE profiles as well as a decline in the DGGE indices, in particular on days 30, 60, and 90. In turn, the XM1 and XM1+Ps or Ps treatments did not affect the values of richness and diversity of the soil bacteria members. A principal component analysis (PCA) also indicated that XM markedly changed the diversity of bacterial assemblages in the second part of the experiment. Moreover, there were differences in the RS/RL of the DGGE indices to the disturbances caused by XM and/or Ps. Considering the mean values of the RS index, the resistance was categorized in the following order: diversity (0.997) > evenness (0.993) > richness (0.970). The soil RL index was found to be negative, thus reflecting the progressing detrimental impact of XM on the genetic biodiversity of bacteria within the experiment. These results indicate that the introduction of XM at higher dosages into the soil environment may exert a potential risk for functioning of microorganism.

## Introduction

Antibiotics and antibiotic-resistant genes are thought to be emerging contaminants that attract considerable public attention due to their potential to harmful effect on the environment and increased risks to human health. They are primarily introduced into soil with sewage sludge, municipal wastewater or animal manures (Kümmerer, 2003; Xia et al., 2005; Chee-Sanford et al., 2009). Recent works have suggested that antibiotics also represent a significant pollution of sediments and soils (Tamtam et al., 2011; de La Torre et al., 2012). This group of pharmaceuticals may enter the fauna, plants and microorganisms, exhibiting a risk to soil organisms and favoring the spread of resistance to antibiotics (Fatta-Kassinos et al., 2011; Gullberg et al., 2011; Brandt et al., 2015).

The second-generation cephalosporins (CPs), active against a wide group of microorganisms, are the most frequently used antibiotics in 20 European countries and represent about 70% of the total outpatient cephalosporin consumption (Versporten et al., 2011). Among this group, cefuroxime (XM) is the most frequently prescribed and, its consumption in Poland and many other European countries constituted more than 50% of the total cephalosporin administration (Coenen et al., 2006; Versporten et al., 2011; Iatrou et al., 2014). Such a high consumption of XM is related to its broad spectrum of antibacterial activity, the resistance to β-lactamase from *Moraxella catarrhalis* and *Haemophilus influenza*, and the activity against *Streptococcus pneumoniae* strains susceptible and resistant to penicillin. XM blocks the synthesis of bacterial cell wall, similarly to antibiotics belonging to the group of β-lactams. It binds with the proteins binding penicillin participated in the synthesis of the peptidoglycan bacterial cell wall causing the lysis of bacteria (Ishibiki et al., 1990; Cheng et al., 2012; Bhattacharya et al., 2015). In the human body, XM is quickly eliminated from the blood and, in unchanged form is nearly completely removed via urine system within 1–3 days (Ishibiki et al., 1990).

The CPs have been found in wastewater and surface water all over the world; however, their highest concentrations were detected in the effluents from hospitals and pharmaceutical industry (Saravanane and Sundararaman, 2009; Oguz and Mihçiokur, 2014; Yu et al., 2016). The concentration of CPs in urban wastewater usually does not exceed 10 μg/L, while their mean concentration in wastewater influent and effluent of CPs producing wastewater is in the range of about 13–142 and 0.1–24 μg/L, respectively. The highest noted concentrations of XM in wastewater influent and effluent reached the values of 210 and 35 μg/L, respectively (Yu et al., 2016). Due to the fact that conventional wastewater treatment plants remove XM from wastewater partially, this antibiotic is introduced into soils through the agricultural usage of sewage sludge. Unfortunately, there is no published data on XM concentrations in soils. However, it may be expected that the introduction of XM into soil may select XM-resistant bacteria and spread the resistance to XM to the bacteria in the environment (Rahube et al., 2014; Luczkiewicz et al., 2015; Kittinger et al., 2016; Devarajan et al., 2017).

Among the many bacteria resistant to antibiotics, some strains of *Pseudomonas putida* have been recognized as increasingly important human pathogens over the last 30 years (Carpenter et al., 2008; Bhattacharya et al., 2015; Fernández et al., 2015; Sun et al., 2016). This opportunistic pathogen is responsible for nosocomial infections, mainly in immunocompromised patients (Yoshino et al., 2011). Outbreaks of the bloodstream infections associated with contaminated fluids have also been observed (Erol et al., 2014; Liu et al., 2014a). *P. putida* is a gram-negative and aerobic bacterium commonly presents in soils. Strains of *P*. *putida* characterize a broad spectrum of biochemical activities related to the ability to degrade various natural and synthetic compounds (Rojas et al., 2001; Espinosa-Urgel et al., 2002; Nelson et al., 2002). In the environment, antibiotic-resistant *P. putida* strains may be participated in the spread of antibiotic-resistant genes among other pathogens (Molina et al., 2014; Sun et al., 2016).

Previously published papers revealed that antibiotics selected antibiotic-resistant bacteria and had an impact on the abundance of soil microorganisms and their activities (Chen et al., 2013; Cui et al., 2013; Liu et al., 2014b; Xu et al., 2016). Moreover, the impact of antibiotics on the genetic diversity and structure of soil microbial communities were reported using the DGGE (Zielezny et al., 2006; Reichel et al., 2013; Cleary et al., 2016; Orlewska et al., 2018) and the phospholipid fatty acid analysis (PLFA) (Demoling et al., 2009; Reichel et al., 2014; Cycon et al., 2016; Xu et al., 2016), respectively. Based on the results related to the activity of other antibiotics, the entry of XM and/or antibiotic-resistant bacteria into soil may also affect the soil bacterial communities. Findings concerning the effect of XM on the genetic diversity of soil bacteria are limited, and therefore, the goal of this research was to check the influence of XM and/or an antibiotic-resistant *Pseudomonas putida* strain on the bacterial community using the DGGE approach and the resistance (RS)/resilience (RL) concept.

## Materials and methods

### Isolation and characterization of the bacterial strain

**Figure 1:**
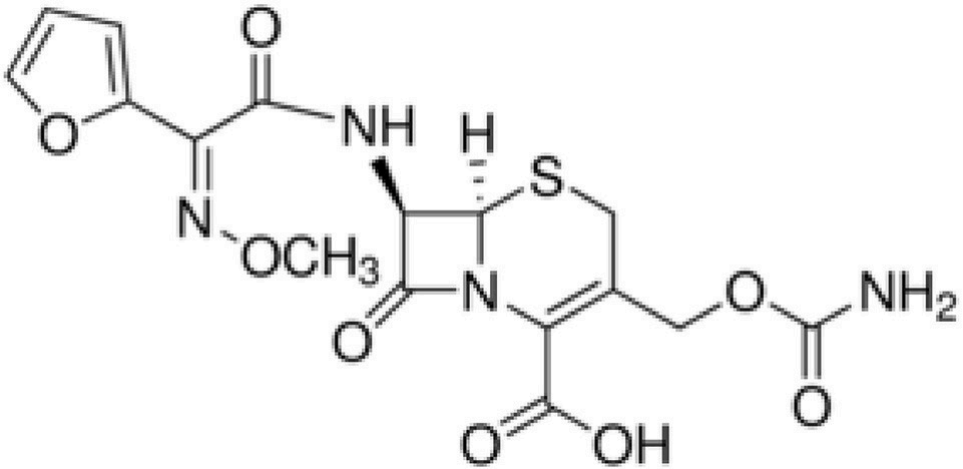
Chemical structure of cefuroxime.

The strain designated as MC1 was isolated from raw sewage on a TSA medium (Tryptone-Soya Agar) with addition of 30 μg XM (Figure 1) (Cycon et al., 2016). Strain MC1 was identified using the 16S rRNA gene analysis with the primers 27f and 1492r (Cycon et al., 2016) and additionally the API 20 NE biochemical test (Cycon et al., 2011). The sequence of strain MC1 was compared to other known sequences of 16S rRNA gene using the BLAST server (NCBI; http://www.ncbi.nlm.nih.gov/). Phylogenetic analysis was performed by the neighbor-joining method using the MEGA ver. 7.0 software. The sensitivity of strain MC1 to XM, clindamycin (CM), ciprofloxacin (CI), erythromycin (EM), vancomycin (VA), tetracycline (TC) or streptomycin (SM) (Table 1) was determined with the use of the disc diffusion and the *E*-test methods (Cycon et al., 2016).

**Table 1:** Results of the sensitivity assays to selected antibiotics for strain MC1.

**Disc-diffusion method**			***E*****-test method**	
**Antibiotic**	**Concentration (μg)**	**Growth inhibition (mm)**	**Range of concentrations (μg/mL)**	**MIC (μg/mL)**
CI	5	28	0.002–32	0.094
CM	2	–	0.016–256	>256
EM	15	–	0.016–256	>256
SM	300	34	0.064–1024	8
TC	30	22	0.016–256	6
VA	30	–	0.016–256	>256
XM	30	–	0.016–256	>256

*CI, ciprofloxacin; CM, clindamycin; EM, erythromycin; SM, streptomycin; TC, tetracycline; VA, vancomycin; XM, cefuroxime; –, no inhibition zone; MIC, minimum inhibitory concentration*.

### Design of the soil experiment

Soil collected from an experimental plot located near the town of Zywiec, Poland and, with the defined characteristics determined with the use of methods described in the ISO standards (Cycon et al., 2010), was used in this experiment. The soil was classified as loamy sand (sand 67%, silt 24%, and clay 9%) with the following main features: pH 6.9, density 1.4 g/cm^3^, water-holding capacity 43%, cation exchange capacity 10 cmol^+^/kg, microbial biomass 932 mg/kg, C_org_ 1.6% and N_tot_ 0.2%.

The experiment included three replications of the following treatments: C (control), XM1 – 1 mg XM/kg soil, XM10 – 10 mg XM/kg soil, Ps – *P*. *putida* MC1, XM1+Ps – 1 mg XM/kg soil + *P*. *putida* MC1 and XM10+Ps – 10 mg XM/kg soil + *P. putida* MC1. Strain MC1 was introduced into soil at 1.6 × 10^7^ cells/g soil and, the preparation of its suspension was made with the use of a previously described method (Cycon et al., 2016). Samples of soil were incubated at 22 ± 1°C and randomly collected during the experimental period for the DGGE analysis.

### Analysis of microbial community structure

The genetic diversity of soil bacteria was analyzed using the amplification of the 16S RNA gene fragment with the primers (GC-clamp)-F338 and R518 (Muyzer et al., 1993) with the use of a previously described method (Cycon et al., 2013). The electrophoresis was run in polyacrylamide gel (8% w/v, 37.5:1 acrylamide:bis-acrylamide) with a linear gradient of denaturant urea (40%−70) using a DCode Mutation Detection System (Bio-Rad, USA). The patterns of the DGGE bands were visualized using a G BOX F3 System (Syngene, UK) (Cycon et al., 2016).

### Data analysis

The patterns of the DGGE bands were evaluated using BioNumerics software ver. 7.5 (Applied Math, Belgium), while the phylogenetic trees were prepared with the use of the unweighted pair-group method and the arithmetic averages (UPGMA) (Cycon et al., 2016). The DGGE indices, i.e., Shannon-Wiener index (H), richness (S) and evenness (E) were calculated using appropriate equations (Cycon et al., 2013). The three-way and two-way ANOVA analyses and the least significant differences (LSD) test (*P* < 0.05) were used to evaluate the obtained results. The data for the DGGE indices were subjected to PCA, and additionally, the PC scores were also evaluated by applying the three-way and two-way MANOVA. Indices adopted from Orwin and Wardle (2004) were applied to assess the resistance (RS) and resilience (RL) of the determined indices to the disturbances caused by the antibiotic and/or bacterial strain. All details of statistical analyzes were presented in a previously published paper (Cycon et al., 2016).

## Results

### Characteristics of the isolate

**Figure 2:**
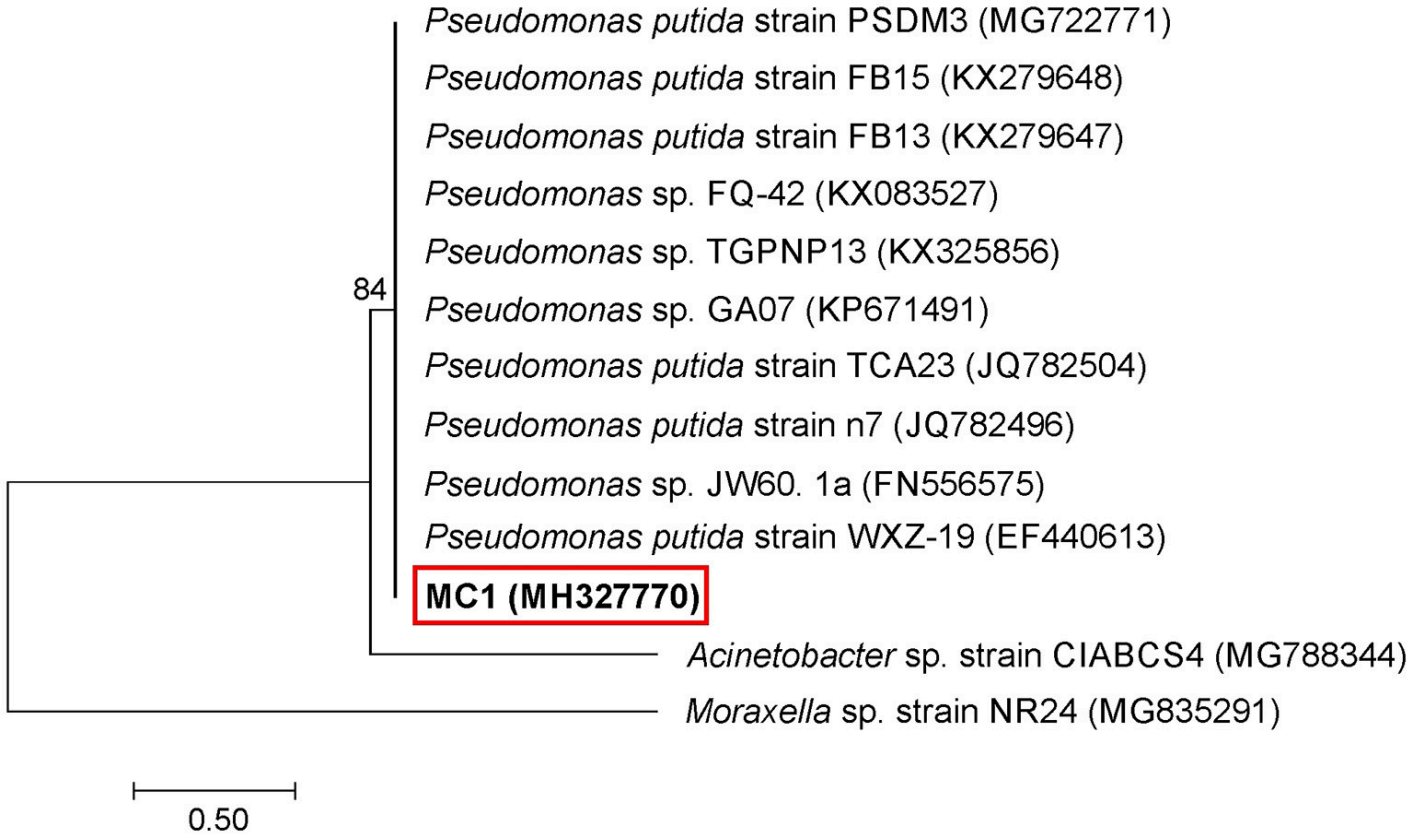
Phylogenetic tree of a multidrug-resistant strain MC1 based on the neighbor-joining method. Bootstrap values from 1,000 replications are indicated at the branches. GenBank accession numbers are given in parentheses.

**Figure 3:**
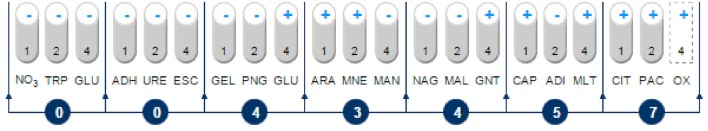
Biochemical pattern and numerical profile of the isolated strain MC1. NO_3_, potassium nitrate; TRP, L-tryptophan; GLU, D-glucose (fermentation); ADH, L-arginine; URE, urea; ESC, esculin; GEL, gelatin; PNG, 4-nitrophenyl-β-D-galactopyranoside; GLU, D-glucose; ARA, L-arabinose; MNE, D-mannose; MAN, D-mannitol; NAG, *N*-acetyl-glucosamine; MAL, D-maltose; GNT, potassium gluconate; CAP, capric acid; ADI, adipic acid; MLT, malic acid; CIT, trisodium citrate; PAC, phenylacetic acid; OX, oxidase.

An analysis of the 16S rRNA gene sequence showed that strain MC1 is a member of the genus Pseudomonas with a high similarity to the species *Pseudomonas putida* (Figure 2). The sequence of strain MC1 was submitted to the GenBank under accession number MC327770. An additional analysis using a biochemical test (Figure 3) also confirmed (98.6% identity) the membership of strain MC1 to the species *P. putida* (numerical profile 0043457). The obtained resistance pattern of strain MC1 to antibiotics showed its resistance to XM, CM, EM and VA as was shown by the MIC values greater than 256 μg/mL (Table 1). In turn, the highest sensitivity of *P. putida* MC1 was noted for SM, TC, and CI, with the MIC values of 8, 6 and 0.094 μg/ml, respectively (Table 1).

### DGGE analysis

**Figure 4:**
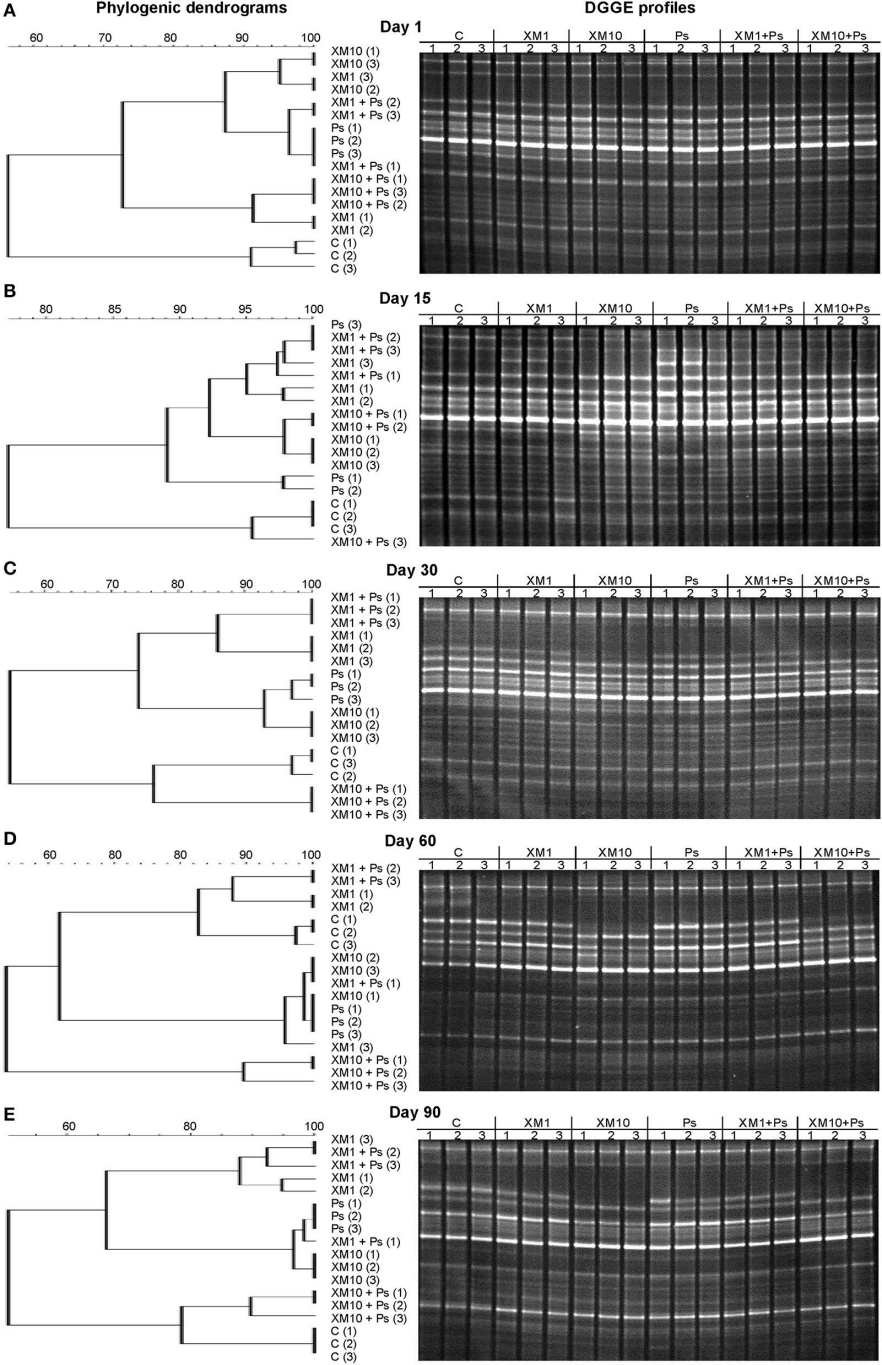
Phylogenic dendrograms based on the DGGE profiles for the soil treated with cefuroxime and/or strain MC1 on days 1 **(A)**, 15 **(B)**, 30 **(C)**, 60 **(D)**, and 90 **(E)**. C, non-exposed soil; XM1, cefuroxime at 1 mg/kg soil; XM10, cefuroxime at 10 mg/kg soil; Ps, *P*. *putida* MC1; XM1+Ps, cefuroxime at 1 mg/kg soil + *P*. *putida* MC1; XM10+Ps, cefuroxime at 10 mg/kg soil + *P*. *putida* MC1.

**Figure 5:**
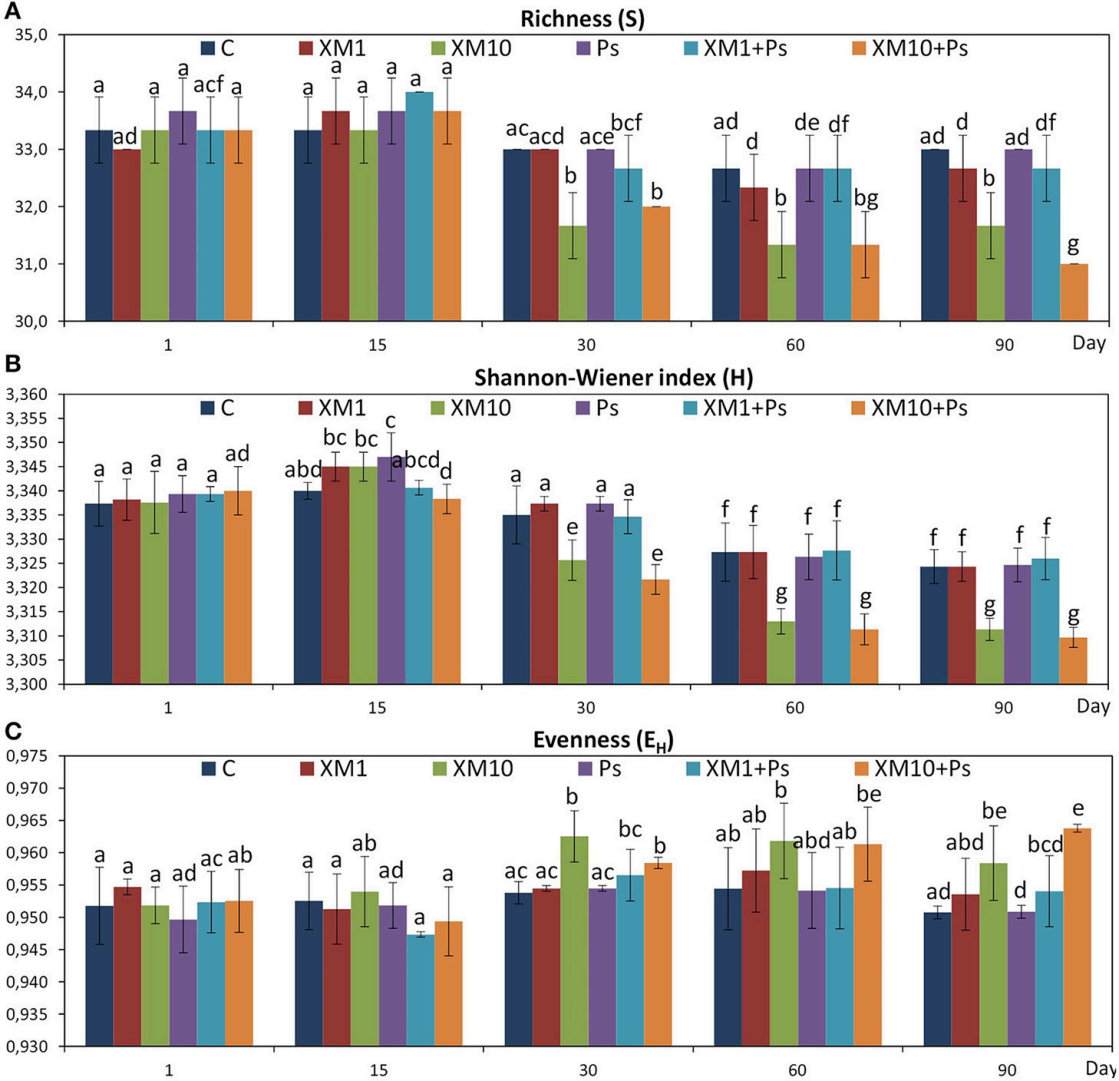
Values of the richness **(A)**, Shannon-Wiener **(B)**, and evenness **(C)** indices for the cefuroxime (XM)- and/or *Pseudomonas putida* MC1 (Ps)-treated soils obtained during the experimental period. The data are the means (*n* = 3) with standard deviations. Different letters within the values of each index indicate significant differences considering the effects of the dose of XM, strain MC1 and incubation time (LSD *post-hoc* test; *P* < 0.05). The explanation of the treatment abbreviations is given in Figure 4.

The obtained results showed that XM affected the composition of the bacterial members in soil microbial community (Figures 4A–E). The DGGE profiles from XM-treated and non-treated soils differed with regards to the absence and density of the bands, in particular on days 30 (Figure 4C), 60 (Figure 4D), and 90 (Figure 4E) of the experiment. The results also revealed that XM at 10 mg/kg (XM10 and XM10+Ps) changed the overall richness (Figure 5A) and diversity (Figure 5B) of the member of bacterial community on days 30, 60, and 90. In contrast, no differences in the S and H values were observed between the lower XM and/or strain MC1 treatments (XM1 and XM1+Ps or Ps) and the non-exposed soil during 90 days. Also, the E_H_ values were generally similar for both treated and non-treated soils on each sampling day (Figure 5C).

The ANOVA revealed that the *S*-value was significantly (*P* < 0.001) affected by the time, the XM dose and the interaction between both factors. The time effect contributed to most of the observed variability (44%) (Table 2). The H index was primarly influenced by the time of incubation (*P* < 0.001), which explained most of the variability (67%) (Table 2). The ANOVA also showed that the concentration of XM and the incubation time were the factors influenced (*P* < 0.01) the E_H_ index within 90 days and contributed to 16 and 21% of the variability, respectively. All details related to the results of the three-way ANOVA are presented in Table 2.

**Table 2:** Analysis of variance (the three-way ANOVA) for the DGGE indices: richness (S), Shannon-Wiener (H) and evenness (E_H_) as affected by the bacterial strain (S), concentration (C), time (T) and their interactions.

**SV**	**S**		**H**		**E**_**H**_	
	**VE**	***P***	**VE**	***P***	**VE**	***P***
S	<1	0.386	<1	0.701	<1	0.400
C	20	<0.001^***^	14	<0.001^***^	16	<0.001^***^
T	44	<0.001^***^	67	<0.001^***^	21	<0.001^***^
S × C	<1	0.827	<1	0.093	<1	0.929
S × T	1	0.481	<1	0.711	2	0.554
C × T	12	<0.001^***^	8	<0.001^***^	11	0.087
S × C × T	1	0.860	1	0.413	3	0.840

SV, source of variance; VE, variance explained (%).

Asterisks represent the significance level according to the ANOVA (^***^P < 0.001).

### Resistance (RS) and resilience (RL) indices

An evaluation of the resistance of the DGGE indices to XM and/or *P*. *putida* MC1 showed that these factors affected the values of the RS index within 90 days (Table 3). In general, the ANOVA revealed that the tested factors had a significant impact (*P* < 0.001) on the resistance of the DGGE indices. The time effect contributed most to the variability in the case of the H index (42%), whereas this effect explained the least of the variability in relation to the E_H_ index (18%) (Table 4). The greatest decrease in the values of the RS index was observed for richness and the mean values of this index for all of the soil treatments were found to be 0.967, 0.965, and 0.950 on days 30, 60, and 90, respectively (Table 3). This decrease was related to the inhibitory effect of XM and/or strain MC1 on the S index (Figure 5A). A similar trend was observed for the H index (Table 3 and Figure 5B). In the case of the E_H_ index, a decrease in the values of the RS index observed for some soil treatments (Table 3) was generally associated with the stimulatory effect of XM and/or strain MC1 during the experimental period (Figure 5C). Taking into account the mean values of the RS index that were calculated for all of the soil treatments during the experimental period, the resistance of the DGGE indices was categorized in the following order: diversity (0.997) > evenness (0.993) > richness (0.970). A calculation of the RL index at the end of the experiment (day 90) revealed that although its value was different for each DGGE index, negative mean values were obtained for all of the soil treatments (Table 5).

**Table 3:** Values of the resistance (RS) index for measured parameters obtained for each day of the experiment.

**Parameter**	**Day**	**Treatment**					**$\bar{x}$**
		**XM1**	**XM10**	**Ps**	**XM1+Ps**	**XM10+Ps**	
Richness (S)	1	0.971^a^	1.000^b^	0.980^abf^	1.000^b^	1.000^b^	0.990^A^
	15	0.980^ac^	1.000^ab^	0.980^acf^	0.961^ce^	0.980^bc^	0.980^AB^
	30	1.000^c^	0.923^d^	1.000^acf^	0.971^e^	0.941^d^	0.967^ABC^
	60	0.980^ac^	0.922^d^	1.000^af^	1.000^ab^	0.922^d^	0.965^BC^
	90	0.971^ae^	0.923^d^	1.000^f^	0.971^e^	0.886^g^	0.950^C^
Shannon-Wiener index (H)	1	0.999^a^	0.999^a^	0.999^af^	0.998^a^	0.998^a^	0.999^A^
	15	0.997^b^	0.997^b^	0.996^b^	1.000^a^	0.999^a^	0.998^AB^
	30	0.998^b^	0.994^c^	0.998^ab^	0.999^ab^	0.992^d^	0.996^B^
	60	1.000^a^	0.991^e^	0.999^af^	1.000^a^	0.990^de^	0.996^B^
	90	1.000^af^	0.992^e^	1.000^f^	0.999^af^	0.991^de^	0.996^B^
Evenness (E_H_)	1	0.992^a^	0.995^a^	0.997^ae^	0.998^af^	0.998^a^	0.996^A^
	15	0.998^a^	0.997^a^	0.998^ae^	0.989^bf^	0.994^ab^	0.995^A^
	30	0.998^a^	0.982^c^	0.998^ae^	0.994^abf^	0.991^bd^	0.993^AB^
	60	0.994^a^	0.985^c^	0.999^ae^	1.000^a^	0.986^cd^	0.993^AB^
	90	0.992^af^	0.985^c^	1.000^e^	0.992^f^	0.974^g^	0.988^B^

*Significant differences (P < 0.05, LSD test) within the mean values (n = 3) of each parameter, considering the effects of the treatment and incubation time, are indicated by different lowercase letters. Significant differences (P < 0.05, LSD test) within the values of all of the treatments for each parameter considering the effect of the incubation time are indicated by different uppercase letters. The values marked in gray or green indicate a significant inhibition or stimulation in relation to the non-treated soil, respectively. The explanation of the treatment abbreviations is given in Figure 4*.

**Table 4:** Analysis of variance (the two-way ANOVA) for the RS indices as affected by the treatment (Tr), time (T) and their interactions.

**SV**	**S**		**H**		**E**_**H**_	
	**VE**	***P***	**VE**	***P***	**VE**	***P***
Tr	27	<0.001^***^	42	<0.001^***^	18	<0.001^***^
T	16	<0.001^***^	11	<0.001^***^	8	<0.001^***^
Tr × T	14	<0.001^***^	40	<0.001^***^	6	<0.001^***^

SV, source of variance; VE, variance explained (%). Asterisks represent the significance level according to the ANOVA (^***^P < 0.001).

**Table 5:** Values of the resilience (RL) index for the measured parameters obtained at the end of the experiment.

**Parameter**	**Treatment**					**$\bar{x}$**
	**XM1**	**XM10**	**Ps**	**XM1+Ps**	**XM10+Ps**	
Richness (S)	0.000^a^	−1.000^b^	1.000^c^	−1.000^b^	−1.000^b^	−0.400
Shannon-Wiener index (H)	0.471^a^	−0.823^b^	0.715^a^	0.139^a^	−0.693^b^	−0.038
Evenness (E_H_)	−0.017^a^	−0.466^bc^	1.000^b^	−0.428^ac^	−0.858^c^	−0.154

*The data are the means (n = 3). Significant differences (P < 0.05, LSD test) within the values of each parameter are indicated by different letters. The explanation of the treatment abbreviations is given in Figure 4*.

### PCA of the DGGE pattern

**Figure 6:**
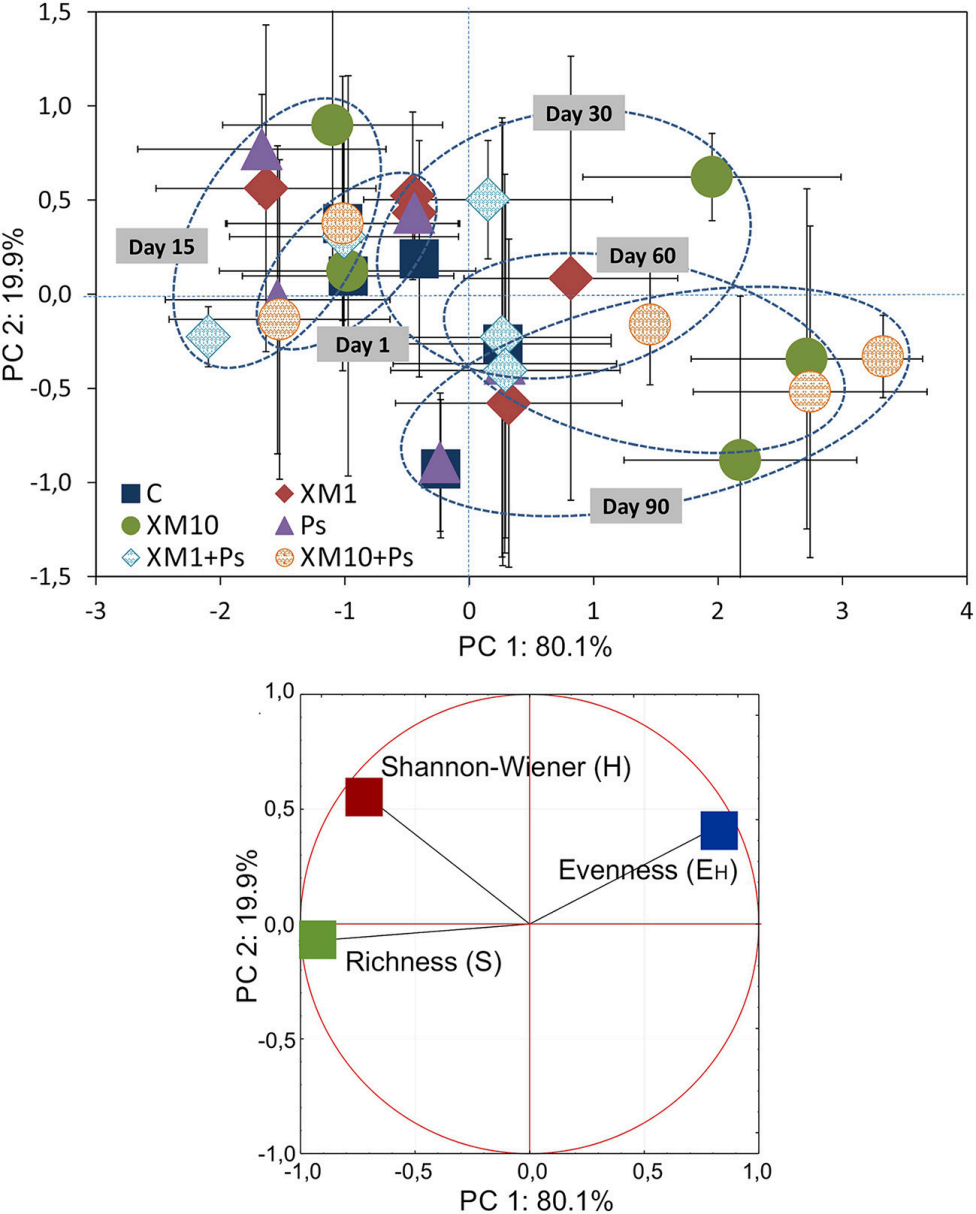
The PC plot for the DGGE indices obtained for all of the sampling days. The explanation of the treatment abbreviations is given in Figure 4.

Based on the PCA of the DGGE indices it was revealed that the introduction of XM and/or *P*. *putida* MC1 altered the pattern of bacterial diversity. The PCA plot created for all days indicated that the treatments were scattered along the PC1 and PC2 axes, which explained 80 and 20% of the total variability, respectively (Figure 6). Also, a three-way MANOVA analysis confirmed this results. The time explained 48 and 28% of the total variance for PC1 and PC2, respectively (Table 6). In turn, the XM concentration explained 20% of the total variance only along PC1. Strain MC1 did not affect the pattern of bacterial biodiversity; it contributed to <1% of the total variance in the PCA plot. All details related to the results of the three-way MANOVA are presented in Table 6.

**Table 6:** Multivariate analysis of variance (the three-way MANOVA) for the PC1 and PC2 based on the data of the DGGE indices for all of the sampling days as affected by the bacterial strain (S), concentration (C), time (T) and their interactions.

**SV**	**PC1**		**PC2**	
	**VE**	***P***	**VE**	***P***
S	<1	0.409	<1	0.429
C	20	<0.001^***^	<1	0.661
T	48	<0.001^***^	28	<0.001^***^
S × C	<1	0.739	1	0.624
S × T	1	0.462	2	0.661
C × T	13	<0.001^***^	3	0.922
S × C × T	1	0.832	5	0.758

SV, source of variance; VE, variance explained (%). Asterisks represent the significance level according to the ANOVA (^***^P < 0.001).

**Figure 7:**
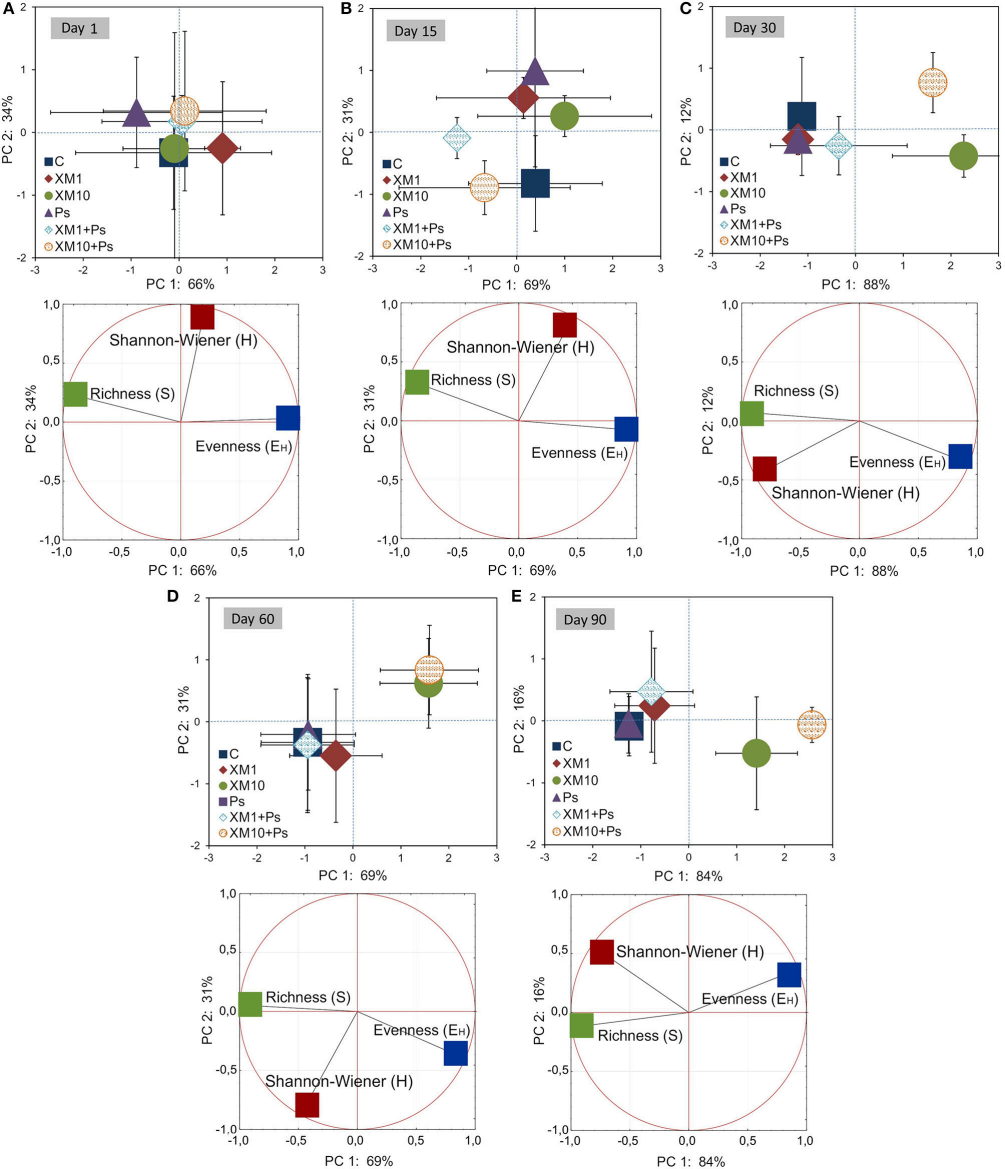
PC plots for the DGGE indices obtained on days 1 **(A)**, 15 **(B)**, 30 **(C)**, 60 **(D)**, and 90 **(E)**. The explanation of the treatment abbreviations is given in Figure 3.

The PCA plots created for each sampling day revealed a meaningful impact of the concentration of XM on the bacterial diversity in the second part of the experiment (Figure 7). The effect of the dose explained 77, 65, and 85% of the variability only along PC1 on days 30, 60, and 90, respectively. On the contrary, no effect of *P. putida* MC1 along PC1 and PC2 was observed on any sampling day. All details related to the results of the three-way MANOVA are presented in Table 7.

**Table 7:** Multivariate analysis of variance (the two-way MANOVA) for the PC1 and PC2 based on the data of the DGGE indices for each sampling day (1, 15, 30, 60 and 90) as affected by the bacterial strain (S), concentration (C), time (T) and their interaction (S × C).

**PC**	**SV**	**1**		**15**		**30**		**60**		**90**	
		**VE**	***P***	**VE**	***P***	**VE**	***P***	**VE**	***P***	**VE**	***P***
PC1	S	3	0.532	13	0.163	<1	0.917	<1	0.693	1	0.235
	C	9	0.561	8	0.525	77	<0.001^***^	65	0.002^**^	85	<0.001^***^
	S × C	3	0.807	7	0.587	4	0.337	1	0.832	3	0.186
PC2	S	8	0.325	<1	0.990	4	0.349	1	0.708	3	0.492
	C	0	0.993	6	0.463	7	0.468	31	0.107	16	0.325
	S × C	0	0.985	49	0.012^*^	35	0.050	<1	0.997	2	0.887

PC, principal component; SV, source of variance; VE, variance explained (%). Asterisks represent the significance level according to the ANOVA (^*^P < 0.05, ^**^P < 0.01, ^***^P < 0.001).

## Discussion

Strain MC1 identified as *Pseudomonas putida* was isolated in this study. *P. putida* was considered as bacteria with low pathogenicity and generally sensitive to antibiotics belonging to different classes (Luczkiewicz et al., 2015; Devarajan et al., 2017). The highest sensitivity of *P. putida* MC1 was determined for CI, SM, and TC. The sensitivity assays to selected antibiotics also showed that the isolated strain MC1 had a multidrug-resistant ability. The disc diffusion and *E*-test methods revealed that this strain was characterized by a resistance not only to XM but also to CM, EM, and VA as was shown by the MIC values greater than 256 μg/ml. Antibiotic-resistant strains of *P. putida* are frequently found in the environment; however, a multidrug-resistant *P*. *putida* has only been isolated from patients in recent years (Lombardi et al., 2002; Bhattacharya et al., 2015; Fernández et al., 2015; Kittinger et al., 2016). The presence of XM in soil may significantly increase the pool of genes responsible for resistance to XM among soil microorganisms. Bacteria can develop resistance to XM and other CPs by producing extended-spectrum lactamases (ESBL), taking up of genes encoding ESBL from soil bacteria, and a high expression of lactamase (*bla*) genes located on chromosome (Pfeifer et al., 2010). The spreading of XM resistance genes may also result from the introduction of XM-resistant bacteria into soil. There is a potential risk that genes responsible for the resistance to XM located on both chromosomes and plasmids may be transferred between mobile genetic elements and spread through horizontal gene transfer (HGT) to autochthonous bacteria. Due to well adaptation of these bacteria to soil conditions, they might be participated in the long-term maintenance of antibiotic resistance genes in soils. HGT may also facilitate the transfer of antibiotic resistance genes from environmental to clinical strains (Pfeifer et al., 2010; Marti et al., 2013).

The application of bacteria resistant to antibiotics into soil may also produce significant changes within the soil microbial assemblages. However, in our study introduction of *P. putida* MC1 did not affect the genetic diversity of the autochthonous microbial communities. This effect might resulted from the competition between the introduced strain and indigenous microorganisms. Furthermore, many compounds produced by autochthonic microorganisms may limit the growth of inoculants (Karpouzas et al., 2000). The lack of any significant changes exerted by strain MC1 in the bacterial diversity may also be connected with the survival of inoculants in the soil environment. Strain MC1 was originated from raw sewage and it probably did not have the capability to adapt to soil conditions and compete with residential microorganisms. In turn, the obtained results indicated that XM may contribute to the modification of the soil microbial diversity. The DGGE band patterns of the soil samples treated with XM differed from the control what resulted from a disappearance of some of the bands in a response to the antibiotic presence, especially on days 30, 60, and 90 of the experiment. Moreover, a decline in the DGGE indices for soils treated with XM was found. There is no reported data concerning the impact of XM and/or *P. putida* on soil microbial diversity and therefore we cannot compare our results with those reported by other authors. However, some previously described results also showed alterations within bacterial communities expressed by the alterations in the number and intensity of bands in a response to the antibiotic application. Shifts in the DGGE band patterns obtained for soil treated with tylosin (TYL) were also noted by Westergaard et al. (2001). The authors revealed that the application of TYL at 2,000 μg/g soil altered the bacterial structure in comparison with the control soil. A lower number of bands in the TYL-exposed soils as compared to the non-treated soil was noted after 2 and 3 weeks of the experiment, however the small changes were seen to the end of the experiment (Westergaard et al., 2001). Additionally, Cycon et al. (2016) revealed that the glycopeptide antibiotic vancomycin (VA) and/or the introduction of VA-resistant *Citrobacter freundii* exerted a selective pressure leading to alterations in the genetic diversity of soil bacterial communities within 90 days. Contrary, Zielezny et al. (2006) noted that the antibiotic sulfadiazine (SDZ) applied at different concentrations (1–50 mg/kg soil) did not affect the bacterial diversity as determined using the PCR-DGGE approach. The authors also revealed that chlortetracycline applied at the same concentrations as sulfadiazine did not change the microbial community structure.

The effect of antibiotics on soil autochthonous bacteria may also be connected with the resilience and resistance of tested microbial communities. The values of the RS and RL indices show whether microbial communities exposed to various stress factors can remain stable and/or achieve the original community structure (Orwin and Wardle, 2004; Orwin et al., 2006). Our experiment showed that the values of the RS index calculated for the DGGE indices for the soils with a higher dosage of XM (XM10, XM10+Ps) were significantly lower than those obtained for the XM1-treated soil. The negative effect of XM applied at a higher concentration on microbial diversity was also proven by the values of the RL index, which were found to be negative, thereby reflecting the progressing detrimental effect of XM on the genetic diversity of bacterial communities during the 90-day experiment. These results showed that in the soils that had been treated with a lower dose of XM and bacteria inoculants, the intrinsic properties of the soil microbial communities were protected the stability of soil ecosystem (Song et al., 2015). Even if some microbial populations were sensitive to the stressor, the whole community was resilient and had the ability to return to its original state (Allison and Martiny, 2008). Such an ability of microbial communities was not found in the XM10-treated soils. The high concentration of XM probably killed a significant part of the microbial population.

Our results showed that the negative effect of XM applied at a higher dosage (XM10 and XM10+Ps) was observed in the second part of the experiment. Although the reason for this phenomenon was not investigated in our study, a harmful effect of XM on a sensitive bacterial population might be connected with the stability and bioavailability of XM in soil and/or intermediates of XM degradation pathways that might also be characterized by antimicrobial properties. The stability of CPs and their susceptibility to degradation strongly depends on environmental conditions and, many biodegradation experiments conducted in various water systems showed that the effectiveness of CP removal varied significantly (Alexy et al., 2004; Gartiser et al., 2007; Jiang et al., 2010). For example, Gartiser et al. (2007) studying the inherent biodegradability of antibiotics using the CO_2_ evolution test found that XM was degraded in activated sludge up to 10% of the initial dosage within 28 days. The middle persistence of XM was also observed in the closed bottle test, in which about 23% of XM remained in the system after 28 days of the experiment (Alexy et al., 2004). The removal rate of about 30% of the initial XM concentration was also observed by Yu et al. (2016) at 25°C during the 144-h experiment. The authors also reported that the persistence of XM was much higher than other tested CPs, i.e., ceftriaxone, cafelexin, and cephazolin. In a study by Jiang et al. (2010), up to 80% of XM was degraded in the lake water and sediments within 168 h. Their results proved that the biodegradation is the main process responsible for the removal of CPs from sediments. Cefuroxime is slowly eliminated from soils and 42.8–80% of the initial dose was degraded within 64 days under aerobic conditions. Other studies also confirmed that the soil properties and additional compounds, such as slurry, manure or sewage, have a significant effect on the bioavailable fraction and degradation of antibiotics in different soils (Hammesfahr et al., 2008; Pan and Chu, 2016; Wang et al., 2016).

Because XM is characterized by a wide-spectrum activity against many bacteria, some of members of a bacterial assemblage in the XM-treated soil were negatively affected. Microorganisms that are sensitive to antibiotics are killed or their number decreases significantly, which results in increased numbers of bacteria resistant to antibiotics. Ding et al. (2014) observed a high disturbance and a low stability of soil bacterial communities in soil contaminated with SDZ (100 mg/kg soil) and manure compared to bacterial communities from soil treated with manure without SDZ. Moreover, numerous taxa such as *Gemmatimonas, Leifsonia, Devosia, Clostridium, Shinella*, and *Peptostreptococcus* containing also human pathogens dominated in the SDZ-amended soil while in the soil with SDZ and manure, the high number of the bacteria from the genera *Hydrogenophaga, Lysobacter, Pseudomonas*, and *Adhaeribacter* that typically are involved in in the maintenance of high soil quality were found (Ding et al., 2014). In another study, SDZ (10 and 100 μg/g soil) applied into soil with manure also altered the bacterial diversity (Hammesfahr et al., 2008). Although the DGGE profiles proved the impact of SDZ+manure on soil bacterial communities on days 32 and 61 after the antibiotic introduction, these effects were not clearly visible for pseudomonads and β-Proteobacteria and may be explained by the resistance of many strains to sulfonamides (Hammesfahr et al., 2008). Genetic changes within the β-Proteobacteria and Pseudomonas group in manure and SDZ-amended soils were also reported by Reichel et al. (2014). Additionally, the presence of XM in soil might cause an overgrowth of fungi that are not susceptible to XM. Such phenomenon was earlier observed for soil treated with sulfadiazine (Hammesfahr et al., 2008), tetracycline (Yang et al., 2009) and oxytetracycline (Chessa et al., 2016).

## Conclusions

The results of our study indicated that the antibiotic-resistant *Pseudomonas putida* MC1 that was introduced into soil did not affect the genetic diversity of the autochthonous microbial communities. In turn, the obtained results showed that XM may cause alterations in the diversity of the soil bacteria. The DGGE patterns from the XM-treated soils differed from the patterns for non-treated control via the dissipation of some bands in a response to the XM application, especially on days 30, 60, and 90 of the experiment as was also evidenced by the decline in the S and H values. Because XM is active against different bacteria, some of members of bacterial communities in the XM-treated soil were negatively affected. The negative effect of XM observed in the second part of the experiment might be related to the stability/bioavailability of XM in the soil and/or more probably from the XM metabolites that are formed, which might also be characterized by antimicrobial properties. Moreover, differences in the resistance and resilience of the DGGE indices to disturbances caused by XM and/or strain MC1 have been demonstrated. The soil RL index was found to be negative, thereby reflecting the progressing detrimental impact of XM on the genetic diversity of soil bacteria within 90 days. These results that the introduction of XM at higher dosages into the soil may exert a potential risk for functioning of microorganism and further disturbances in the soil activity.

## Author contributions

KO and MC conceived and designed the experiments. KO contributed the reagents and materials. KO and MC performed the experiments. KO, MC, and ZP-S analyzed the results. KO, MC, and ZP-S wrote the paper.

### Conflict of interest statement

The authors declare that the research was conducted in the absence of any commercial or financial relationships that could be construed as a potential conflict of interest.
